# Preventive effect of goby fish protein hydrolysates on hyperlipidemia and cardiovascular disease in Wistar rats fed a high-fat/fructose diet

**DOI:** 10.1039/c7ra13102j

**Published:** 2018-03-06

**Authors:** Rim Nasri, Ola Abdelhedi, Ines Jemil, Ikram Ben Amor, Abdelfattah Elfeki, Jalel Gargouri, Ahmed Boualga, Maha Karra-Châabouni, Moncef Nasri

**Affiliations:** Laboratory of Enzyme Engineering and Microbiology, University of Sfax, National School of Engineering of Sfax (ENIS) P. O. Box 1173 Sfax 3038 Tunisia abd.ola1502@gmail.com +216 74 275 595 +216 74 274 088; Laboratory of Animal Ecophysiology, University of Sfax, Faculty of Sciences of Sfax (FSS) P. O. Box 95 Sfax 3052 Tunisia; Laboratoire de Nutrition Clinique et Métabolique, Faculté des Sciences, de la nature et de la vie, Université d'Oran 1 Ahmed Ben Bella Oran Algeria; Centre Régional de Transfusion Sanguine de Sfax, Route El-Ain Km 0.5 CP 3003 Sfax Tunisia

## Abstract

This study was carried out to investigate the hypolipidemic, cardioprotective and anticoagulant properties of fish goby protein hydrolysates (GPHs) in rats fed a high fat and fructose diet (HFFD). Wistar rats were fed with HFFD for 2 months, coupled with the oral administration of GPHs and undigested goby protein (UGP). Compared with the standard diet, HFFD induced dyslipidemia and liver structure alterations, and increased pancreatic lipase activity. In addition, HFFD caused a significant increase in body weight. Interestingly, administration of UGP and GPHs to HFFD fed rats was efficacious in lowering serum total cholesterol (TC), triglyceride (TG) and low-density lipoprotein cholesterol (LDL-c) as well as hepatic TC and TG, and increased the serum high density lipoprotein cholesterol (HDL-c) content. Moreover, all treatments significantly decreased the atherogenic index and coagulant factor levels (thrombin and prothrombin). UGP and GPH administration also significantly decreased pancreatic lipase activity, which mitigates lipid accumulation. Similarly, UGP and its hydrolysates showed cardioprotective potential revealed by decreasing the risk of atherogenic and coronary artery disease and improving the liver architecture. The *ex vivo* plasma clotting test showed that GPHs exert a great therapeutic anticoagulant potential. The overall results demonstrated that GPH supplementation can counteract high-fat/fructose diet-induced obesity.

## Abstract 2

This study was carried out to investigate the hypolipidemic, cardioprotective and anticoagulant properties of fish goby protein hydrolysates (GPHs) in rats fed a high fat and fructose diet (HFFD).

## Graphical abstract

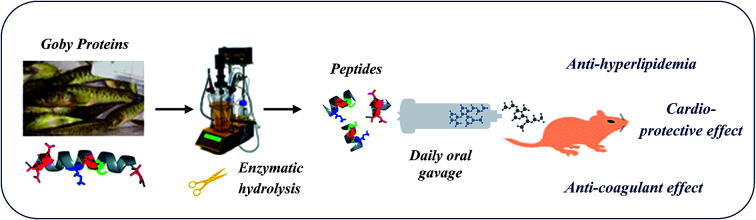

## 1. Introduction

Nutrition represents a principal lifestyle element that can have a direct effect on health. In fact, the long-term positive energy balance between ingested and expended calories leads to the development of obesity or being overweight,^1^ one of the major public health problems throughout the world.^2^ Obesity is defined as a complex disorder involving an excessive accumulation of fat, which may have a negative effect on health, leading to the increase of weight and dyslipidemia. Furthermore, the excessive accumulation of adipose tissue accelerates the development of cardiovascular problems, which constitutes the leading cause of mortality of the obese. The influence of a hypercaloric diet on the development of cardiovascular disease has been reported in several studies.^3,4^ In fact, the annual sudden cardiac death rate was nearly 40 times higher in obese people than in the non-obese population.^5^ The hypercaloric diet like high fat fructose diet promotes the development of various chronic pathologies such as dyslipidemia, hypertension, type 2 diabetes and venous thrombosis.^6^ The continued consumption of high fat diets, especially which are enriched with cholesterol and animal fats represents the major cause of hyperlipidemia, hepatic lipid accumulation, lipid peroxidation and hepatotoxicity.^7^ All these pathological diseases are clustered in a medical disorder constituting metabolic syndrome.^8^ Moreover, epidemiological studies have shown that consumption of high-fat and high fructose diets is correlated with high rates of overweight, central obesity and metabolic syndrome.^9–11^ In addition, further studies have reported that the high fructose-rich diet are usually associated with cardiac alterations by inducing excessive body weight gain, hyperlipidemia, glucose intolerance, hyperinsulinemia, oxidative stress and even atherosclerosis.^12,13^

Commonly, statins represent the most widely prescribed hypolipidemic drugs in the world, which is intended for reducing the cholesterol levels in the blood by inhibiting the enzyme HMG-CoA reductase. They have been currently used to treat hypercholesterolemia in order to prevent cardiovascular diseases.^14^ However, they have a number of undesirable side-effects including muscle damage and skeletal muscle disorder^15,16^ and abnormalities in liver enzymes tests^17^ and increases the risk of diabetes.

Besides, in the field of food science, scientific studies have focused on the search of natural sources (such as phenolic component, caroténoide, ellagitannin).^18–21^ These natural molecules could have a potential effect to suppress the accumulation of body fat,^22^ lipase activity,^23,24^ appetite,^25^ which leads to the endothelial dysfunction and the increase of the atherogenic risk associated with an increase of the thrombotic risk.

Recently, a great deal of interest has been expressed regarding marine-derived bioactive peptides because of their numerous health benefits.^26^ It is well known that oral bioavailability of therapeutic peptide depends on their ability to cross the intestinal mucosa and reach the systemic circulation.^27^ In spite of intestinal poor permeability, peptides of different sizes can pass through the intestinal epithelium by active or passive (paracellular or transcellular) transport processes.^28^ Nevertheless, the small quantities that can penetrate intestinal barrier can exert their biological activity.^29^ In addition, these transport processes can be enhanced by their lipidization with fatty acids which increase peptides lipophilicity. The mechanism by which these FA is their ability to open tight junctions, binding to fatty acid binding proteins as well as solubilising membranes.^30^

Few works investigated the anti-dyslipidemia and anticoagulant activities of marine peptides *in vivo*. Therefore, due to their richness with bioactive peptides and essential amino acids, protein hydrolysates from fish products deserve much interest for their potential effects in reducing cholesterolemia and improving plasma lipid metabolism.^31–33^ The protein, which is an active nutrient present in fish, could influence lipid metabolism and have an interesting cardioprotective effect. Demonty *et al.*^34^ showed the protective effect of cod protein against the hypercholesterolemia by decreasing of serum TC level and reducing of the insulin-resistance in rat fed a high cholesterol diet. Wergedahl *et al.*^35^ reported that the salmon protein tends to decrease the plasma TC level and to increase the ratio C-HDL/TC in the Zucker rat.

Our previous studies demonstrated that goby protein hydrolysates, obtained using different enzymatic preparations, possess anticoagulant,^36^ antioxidant^37^ and anti-ACE^37^*in vitro* activities. Therefore, the aims of this investigation were to investigate the potential of GPHs to regulate dyslipidemia and cardiovascular disorder on rats fed high fat/fructose diets.

## 2. Materials and methods

### 2.1. Materials

Goby (*Zosterissessor ophiocephalus*) and triggerfish (*Balistes capriscus*) were freshly purchased from the fish market of Sfax City, Tunisia. The samples were packed in polyethylene bags and transported to the research laboratory within 30 min. Upon arrival, muscles from goby were separated, rinsed with cold tap water and then with cold distilled water in order to remove salts and other contaminants. They were used immediately or stored in sealed plastic bags at −20 °C until they were used for protein hydrolysates production less than 1 week later. The viscera of triggerfish were used immediately for the extraction of alkaline digestive proteases.

### 2.2. Preparation of proteolytic enzymes

Enzyme preparation from *Bacillus mojavensis* A21 was prepared in our laboratory as reported by Hmidet *et al.*^38^ Regarding the digestive enzymes from triggerfish, 150 g of viscera were thoroughly washed with distilled water and then homogenized for 1 min with 300 ml of extraction buffer (10 mM Tris–HCl, pH 8.0). The homogenate was centrifuged at 8.500 rpm for 30 min at 4 °C. The pellet was discarded, and the supernatant, referred to the protease extract, was collected and used as crude alkaline protease extract.

Protease activity was determined according to the method of Kembhavi *et al.*^39^ using casein as a substrate under standard conditions. One unit of protease activity was defined as the amount of enzyme required to liberate 1 μg of tyrosine per minute under the experimental conditions used.

### 2.3. Preparation of undigested goby protein and its hydrolysates

Raw muscle from goby fish (500 g) in 500 ml distilled water was cooked for 20 min at 90 °C. The bones were removed from cooked fish and fillets were collected, homogenized and freeze-dried to obtain fine powder used as UGP.

Regarding protein hydrolysates, goby muscle (500 g), in 500 ml distilled water, was first minced using a grinder (Moulinex Charlotte HV3, France) then cooked for 20 min at 90 °C to inactivate endogenous enzymes. The cooked muscle sample was then homogenized in a Moulinex® blender for about 5 min. The samples were adjusted to optimum pH and temperature for both enzymes (pH 10.0, 50 °C). The protein solutions were allowed to equilibrate for 30 min before hydrolysis was initiated. Then, the substrate proteins were digested with enzymes at a 1 : 3 (U mg^−1^) enzyme/protein ratio under optimum pH and temperature conditions. Enzymes were used at the same activity levels to compare hydrolytic efficiencies. During the reaction, the pH of the mixture was maintained at the desired value by continuous addition of 4 N NaOH solutions. After incubation, the reactions were stopped by heating the solutions for 20 min at 80 °C to inactivate enzymes. Protein hydrolysates were then centrifuged at 8500 rpm for 20 min. Finally, the soluble fractions, referred to as protein hydrolysates, were freeze-dried using freeze-dryer at a temperature of −50 °C and a pressure of about 121 mbar through a lyophilizer lab (Moduloyd-230, ThermoFisher Scientific, USA) and then stored at −20 °C for further use. GPHs obtained by treatment with protease preparation from *B. mojavensis* A21 and crude protease extract from triggerfish were referred as GPH-A and GPH-TF, respectively.

The degree of hydrolysis (DH), defined as the percent ratio of the number of peptide bonds cleaved to the total number of peptide bonds in the substrate studied, was calculated from the amount of base (NaOH) added to keep the pH constant during the hydrolysis as described by Adler-Nissen.^40^

### 2.4. Amino acid composition

The amino acid compositions were determined as previously reported by Nasri *et al.*^37^

### 2.5. Treatment of animals and diet

Male Wistar rats weighing 110–150 g were purchased from the breeding center of the Central Pharmacy of Tunis (SIPHAT, Tunisia). Animal maintenance and experimental procedures were performed in accordance with the Guidelines for Care and Use of Laboratory Animals of Tunis University and approved by the Animal Ethics Committee of National Institute of Health (1985).^41^ Animals were kept in an environmentally controlled breeding room (temperature: 22 ± 2 °C, relative humidity 60 ± 5%, 12 h dark/12 h light cycle) in the laboratory of the Faculty of Sciences of Sfax, Tunisia. All rats were allowed free access to tap water and alimentation during the experimental period.

The rats were divided into six groups, each containing six animals:

Group CD: rats fed a standard diet and received by gavage 1 ml of tap water during 10 weeks. (Control diet group). The composition of the standard diet is illustrated in the Table 1. Standard diet was supplied by Society of Animals Nutrition, Sfax, Tunisia.

**Table 1:** Composition of experimental diet of corn, soya, VMC (vitamins and mineral compounds) as the following characteristics

		Control diet	HFFD
Nutritional properties (%)	Moisture	14	14
	Fibers	3.4	3.4
	Proteins	22	22
	Fat	3.5	13.5
	Ash	6.7	6.7
	Fructose	0	5
	Caloric value (kcal kg^−1^)	2850	3850
Amino acids (%)	Met	60	60
	Cys	0.38	0.38
	Thr	0.80	0.80
	Trp	0.30	0.30
Mineral mix (mg kg^−1^)	Manganese	80	80
	Iron	48	48
	Cooper	18.75	18.75
	Zinc	65	65
	Selenium	0.30	0.30
	Cobalt	0.20	0.20
	Iodine	1.20	1.20
Vitamin and antioxidant (mg kg^−1^)	Vitamin A	13 000	13 000
	Vitamin D3	4375	4375
	Vitamin H	62.5	62.5
	Antioxidant (BHA-BHT)	125	125

Group HFFD: rats fed fat and fructose enriched diet (HFFD) and received by gavage 1 ml of tap water during 10 weeks. HFFD was prepared by adding 10 g fat, 5 g fructose and 0.1 g cholic acid per kg to standard diet. Cholic acid was included because it increases micelle formation and facilitates intestinal absorption of cholesterol.

Group HFFD + FLUV: rats fed a HFFD and received daily by gavage fluvastatin (20 mg per kg of body weight daily) during 10 weeks.

Groups HFFD-UGP, HFFD-A and HFFD-TF: rats fed HFFD and received by gastric gavage 400 mg per kg of body weight (BW) daily for a period of 10 weeks of UGP, GPH-A, and GPH-TF, respectively. Food intakes of each group were recorded daily, and their body weights were monitored twice a week throughout the experiment. At the end of the experiment, after overnight fasting (12 h), the rats were sacrificed by decapitation.

### 2.6. Blood and tissue collection

At the end of the experimental period (10 weeks), Wistar rats were sacrificed by decapitation to avoid stress. Blood samples were collected for various estimations. Livers from rats were carefully removed, weighed and then stored at −80 °C until use.

Samples of liver tissues were homogenized for 5 min in TBS buffer (50 mM Tris–HCl, 150 mM NaCl, pH 7.4), with a ratio of (1 : 2) (w/v), using an Ultra-Turrax homogenizer. The homogenates were then centrifuged at 9000 rpm for 15 min at 4 °C and the supernatants were collected and used for biochemical estimations.

### 2.7. Determination of plasma and liver biomarkers

Total cholesterol, triglycerides, and high-density lipoprotein cholesterol (HDL-c) concentrations in serum were determined by enzymatic colorimetric methods using available commercial kits (Biomaghreb, Tunisia). Serum TC level was measured using the cholesterol oxidase assay, while serum TG level was measured by the glycerol kinase assay. The resulting coloration intensity was measured at 505 nm and the results were expressed as mmol l^−1^. Despites, the results of hepatic TC and TG levels were expressed as μmol per g of liver tissue. The low-density lipoprotein cholesterol (LDL-c) and the very low-density lipoprotein cholesterol (VLDL-c) fractions were calculated according to the following equations:^42,43^LDL-c = TC − HDL-c − (TG/5)VLDL-c = TG/5

To predict atherosclerosis and cardiovascular problems, atherogenic index (AI), atherogenic index of plasma (AIP) and coronary risk index (CRI) were determined referring to the equations given below:^42,44,45^AI = LDL-c/HDL-cAIP = log(TG/HDL-c)CRI = TC/HDL-c

Pancreatic lipase activity was measured with commercial kit from BIOLABO, France.

### 2.8. Determination of antithrombotic/anticoagulant activity (*ex vivo*)

#### 2.8.1. Activated partial thromboplastin time (aPTT) assay

For the aPTT assay, 50 μl of citrated platelet poor plasma from the experimental rats were incubated for 3 min at 37 °C. Fifty microliters of APTT reagent (CK-PREST, sigma stago) was added and the mixture was then incubated for 3 min at 37 °C. The clotting time was immediately recorded following the addition of 100 μl of 25 mM CaCl_2_. The clotting time is expressed in seconds.

#### 2.8.2. Prothrombin time assay

For the Prothrombin time assay, plasma rat and thromboplastin reagent (sigma stago) were pre-incubated during 3 min at 37 °C. Then, 100 μl of citrated platelet poor plasma rat were added to the tube containing 200 μl thromboplastin reagent, and the clotting time was recorded.

### 2.9. Histological evaluation

Samples from liver of the different groups of rats were fixed in a Bouin solution for 24 h for histological examination, and then transported in a 10% formol solution. The fixed tissues were embedded in parafilm and then sectioned at 4 μm thick. Sections were then stained with hematoxylin–eosin for histological examination.

### 2.10. Statistical analysis

Results were expressed as means ± SEM (Standard Error Mean) and analyzed using the Statistical with SPSS ver. 17.0, professional edition. A one-way analysis of variance (ANOVA) was then performed and followed by Duncun's test to estimate the significance among the main effects at the 5% probability level. Significant differences (*P* < 0.05) between means were identified by multiple comparisons across the six groups using least significant difference (LSD) procedures.

## 3. Results and discussion

### 3.1. Amino acid composition of UGP and GPHs

Biological activities of protein hydrolysates, obtained by enzymatic hydrolysis, strongly depend on several factors, including the specificity of proteases used for hydrolysis and processing conditions. Therefore, in the present study, two protein hydrolysates were prepared by treatment with proteases from *B. mojavensis* A21 (GPH-A), and proteases from triggerfish (GPH-TF). The degree of hydrolysis of GPH-TF and GPH-A were 23.38% and 13.42%, respectively.

The amino acids compositions of undigested goby proteins and its hydrolysates, expressed as residues per 100 residues, are presented in Table 2. Hydrolysis with the proteolytic enzymes changes slightly the percentages of several amino acid residues of the hydrolysates, which may be attributed to the differences in specificity of the enzymes. Gly was the most abundant amino acid which accounted for 15.19% and 15.12% in GPH-TF and GPH-A, respectively, followed by Thr. GPHs have a high percentage of essential amino acids (Val, Met, Lys, Ile, Leu, Phe and Thr).

**Table 2:** Amino acids composition (%) of UGP and GPHs

Amino Acids	UGP	GPH-TF	GPH-A
Asx	4.41	3.74	3.95
Ser	7.02	6.54	6.80
Glx	9.20	7.94	7.8
Gly	15.74	15.19	15.12
His^a^	3.62	3.93	4.05
Arg	8.76	8.68	6.88
Thr^a^	13.40	12.55	12.96
Ala^b^	6.20	5.86	6.08
Pro^b^	4.25	5.28	5.20
Cys^b^	3.20	1.61	2.54
Tyr^a^^,^^b^^,^^d^	2.21	3.63	3.55
Val^b^^,^^c^	3.50	4.71	4.31
Met^a^^,^^b^	1.99	2.24	2.84
Lys^a^	4.25	3.99	4.00
Ile^a^^,^^b^^,^^c^	2.36	3.31	2.96
Leu^a^^,^^b^^,^^c^	6.84	6.69	6.42
Phe^a^^,^^b^^,^^d^	3.00	4.01	4.52
aEAA	37.67	42.28	41.84
bAAH	28.69	41.33	38.42
cBCAA	12.70	14.71	13.69
dAAA	5.21	7.64	8.07

a EAA: essential amino acids.

b HAA: hydrophobic amino acids.

c BCAA: branched-chain amino acids.

d AAA: aromatic amino acids. GPH-A and GPH-TF are goby protein hydrolysates produced using bacterial proteases of *Bacillus mojavensis* A21 and digestive enzymes from triggerfish (*Balistes capriscus*), respectively. UGP is the undigested goby proteins.

### 3.2. Effects of UGP and GPHs on organs weights, body weight gain and food intake

It is of interest to mention that no toxic effects were observed when the rats were administered with the samples. Further, the behavior of the animals was normal throughout the whole experiment.

**Fig. 1:**
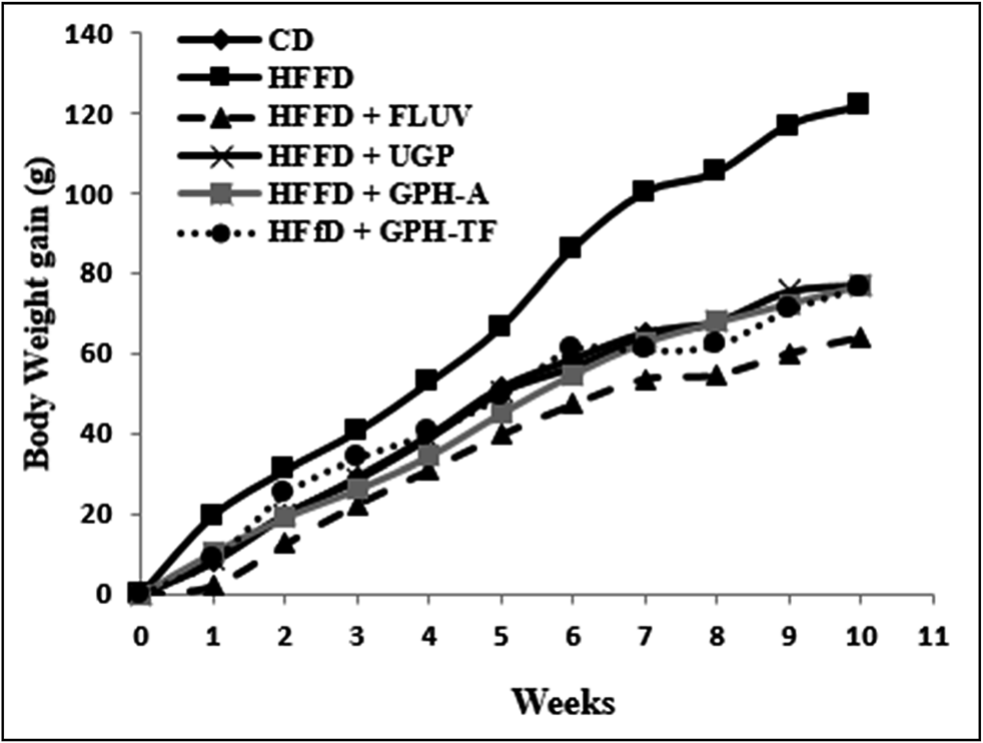
Effects of UGP and GPHs administration on body weight gain (g) in rats fed a high-fat and fructose diet. GPH-A and GPH-TF represent goby protein hydrolysates produced using bacterial proteases of *Bacillus mojavensis* A21 and digestive enzymes from triggerfish (*Balistes capriscus*), respectively. UGP is the undigested goby proteins. Data are mean ± SEM (*n* = 6).

The body weight gains (g) of the experimental groups were determined and reported in Fig. 1. Compared with control rats, which fed standard diet, the BW gain after ten weeks of the HFFD group increased significantly (*p* < 0.05) by 67.97%, demonstrating the role of fat and fructose enriched-diet, which provided a higher calorie intake than normal diet and created an imbalance of energy balance in untreated group. Orally administration of UGP, GPH-A and GPH-TF reduced clearly (*p* < 0.05) overweight when compared to the HFFD group, and its similar to the normal rats. The effect of undigested proteins and its hydrolysates were more notable than that of FLUV.

**Fig. 2:**
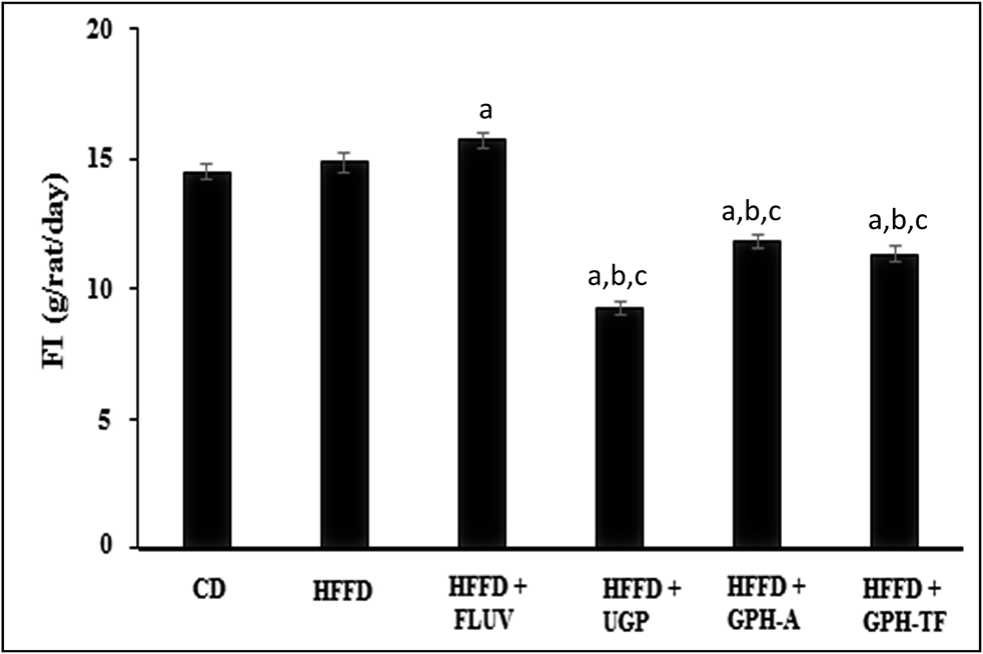
Effects of UGP and GPHs administration on daily food intake (FI) of rats fed a high-fat and fructose diet. GPH-A and GPH-TF represent goby protein hydrolysates produced using bacterial proteases of *Bacillus mojavensis* A21 and digestive enzymes from triggerfish (*Balistes capriscus*), respectively. UGP is the undigested goby proteins. Data are expressed as mean ± SEM (*n* = 6). ^a,b,c^ Indicate significant differences compared to the values of CD, HFFD and HFFD + FLUV groups, respectively, at *p* < 0.05.

The daily food intakes (FI) among the experimental groups are shown in Fig. 2. The daily FI of the HFFD group was significantly higher than that of the control group (*p* < 0.05). Interestingly, UGP and GPH-A and GPH-TF remarkably decreased the FI by 37.66%, 20.44% and 23.80%, respectively, compared to that of HFFD group. UGP was more efficient than its hydrolysates in the decrease of FI. No significant difference was observed between groups treated with the two GPHs (*p* > 0.05). The decrease of FI could be attributed to the fact that UGP and its hydrolysates might exert appetite-suppressing effect in rats. Cudennec *et al.*^25^ reported that protein hydrolysates from blue whiting muscle have satiating properties by reducing the short-term food intake, which was correlated with increasing cholecystokinin peptide and glucagon-like peptide-1 plasma levels. In fact, these peptides, released in intestinal lumen, bind their receptors in hypothalamus inducing the liberation of orexigenic neuropeptides and the feeling of satiety. Moreover, the lowest weight gain that is noted in GPH-FT may be due to a potent stimulation of CCK secretion by the low size of peptides presents in GPH-FT compared with GPH-A (that hydrolyzed respectively at DH 23% and 13%). In the same way, Caron *et al.*^46^ showed that increased secretion of anorexigenic CCK related to the size of the peptide. So the rich fractions in peptides below 1000 Da appeared to be the most potent for stimulating CCK secretion.

Table 3 shows the weights of rats' organs (the ratio of organ to final body weight) at the end of the experiment. There were no significant differences in the weight of the different organs among the experimental groups, indicating that the administration of GPHs had no toxic effect at the experimental dose.

**Table 3:** Effects of UGP and GPHs on relative organs weights of rats fed HFFD^a^

Relative organ weight (g/100 g of body weight)	CD	HFFD	HFFD + FLUV	HFFD + UGP	HFFD + GPH-A	HFFD + GPH-TF
Heart	0.40 ± 0.03	0.39 ± 0.07	0.33 ± 0.03	0.40 ± 0.03	0.35 ± 0.02	0.38 ± 0.04
Pancreas	0.25 ± 0.04	0.32 ± 0.05	0.27 ± 0.01	0.21 ± 0.02	0.29 ± 0.06	0.24 ± 0.03
Kidneys	0.77 ± 0.05	0.67 ± 0.02	0.67 ± 0.07	0.61 ± 0.08	0.59 ± 0.08	0.70 ± 0.04
Liver	3.14 ± 0.34	2.95 ± 0.22	2.72 ± 0.27	3.51 ± 0.22^c^	3.29 ± 0.21	3.30 ± 0.25

a GPH-A and GPH-TF are goby protein hydrolysates produced using bacterial proteases of *Bacillus mojavensis* A21 and digestive enzymes from triggerfish (*Balistes capriscus*), respectively. UGP is the undigested goby proteins. Data are expressed as mean ± SEM (*n* = 6).

### 3.3. Effects of UGP and GPHs on plasma lipid profiles

The levels of serum lipids in the different groups at the end of the experiment are shown in Table 4. As expected, HFFD caused hyperlipidemia. Indeed, HFFD resulted in a significant increase (*p* < 0.05) in the serum TG, TC, LDL-C and VLDL levels by approximately 78%, 72%, 250% and 78%, respectively, compared to those of the control group (Table 4). High levels of TG, TC and LDL-c and low level of HDL-c in serum are strongly associated with increased risk of cardiovascular disease. These results are consistent with those of Ghibaudi *et al.*^47^ who indicate that higher levels of dietary fat increase the susceptibility to diet-induced obesity in growing rats by stimulating caloric intake, fat accretion, and weight gain and by increasing plasma levels of glucose, cholesterol, triglycerides. These deleterious effects are strongly associated with increased risk of cardiovascular disease.

**Table 4:** Effects of UGP and GPHs on the serum levels of TC, TG, HDL-C, LDL-C, and on Atherogenic Index (AI), Atherogenic Index of plasma (AIP) and Coronary Risk Index (CRI)^a^

	CD	HFFD	HFFD + FLUV	HFFD + UGP	HFFD + GPH-A	HFFD + GPH-TF
TG	1.27 ± 0.16	2.26 ± 0.13^a^	1.80 ± 0.06^a,b^	1.52 ± 0.11^b^	2.10 ± 0.16^a^	1.24 ± 0.15^b,c^
TC	1.34 ± 0.01	2.30 ± 0.09^a^	1.80 ± 0.06^a,b^	2.02 ± 0.07^a^	2.01 ± 0.08^a,b^	1.77 ± 0.15^a,b^
HDL-c	0.55 ± 0.01	0.52 ± 0.02	0.69 ± 0.02^a,b^	0.70 ± 0.01^a,b^	0.77 ± 0.02^a,b,c^	0.74 ± 0.04^a,b^
LDL-c	0.22 ± 0.01	0.77 ± 0.05^a^	0.29 ± 0.07^b^	0.62 ± 0.06^a,c^	0.27 ± 0.02^b^	0.47 ± 0.08^a,b,c^
VLDL-c	0.58 ± 0.07	1.03 ± 0.06^a^	0.82 ± 0.03^a,b^	0.69 ± 0.05^b^	0.95 ± 0.07^a^	0.56 ± 0.07^b^
AI	0.40 ± 0.02	1.44 ± 0.08^a^	0.42 ± 0.10^b^	0.90 ± 0.10^a,b,c^	0.35 ± 0.02 ^b^	0.63 ± 0.09^b^
AIP	0.39 ± 0.05	0.63 ± 0.01^a^	0.41 ± 0.03^b^	0.34 ± 0.06^b^	0.43 ± 0.03^b^	0.22 ± 0.01^a,b,c^
CRI	2.45 ± 0.01	4.43 ± 0.05^a^	2.60 ± 0.05^b^	2.89 ± 0.14^a,b,c^	2.58 ± 0.09^b^	2.40 ± 0.12^b^

a GPH-A and GPH-TF represent goby protein hydrolysates produced using bacterial proteases of *B. mojavensis* A21 and digestive enzymes from triggerfish (*Balistes capriscus*), respectively. UGP is the undigested goby proteins. Data are expressed as mean ± SEM (*n* = 6). ^a,b,c^ Indicate significant differences compared to the values of CD, HFFD and HFFD + FLUV groups, respectively, at *p* < 0.05. AI = LDL/HDL; AIP = log(TG/HDL); CRI = TC/HDL.

In the present study, the daily administration GPHs and FLUV improved lipid profile revealed by a decrease of TC, TG, LDL-c and VLDL-c levels. These effects can be attributed to the low energy intake. The hypolipidemic effect of GPH-TF was greater than GPH-A and even better than FLUV. Although UGP showed hypolipidemic effect it was less efficient than its hydrolysates. The obtained results demonstrate the importance of *in vitro* protein hydrolysis.

The reduction of serum TC in treated HFFD rats can be mostly due to a decline of VLDL-c, LDL-c and improvement of HDL-c concentrations. The mechanisms, by which UGP and GPHs prevented hypercholesterolemia, were not yet established. However, some hypotheses may be advanced to explain this cholesterol-lowering effect. In fact, this lowering effect observed particularly with GPH-TF could be explained by a decrease 3-hydroxyl-3-methyl-glutaryl-coenzyme A (HMG CoA) reductase activity which caused a reduction in cholesterol biosynthesis.^48^ The second explanation could be suggested that GPHs increase cholesterol uptake from circulation by facilitating the uptake of VLDL and their remnant *via* LDL receptor-related protein. These results were consistent with those of Lassoued *et al.*,^32^ Ben Khaled *et al.*^31^ and those of Liaset *et al.*^49^ who found that rats fed the fish protein hydrolysate had increased VLDL receptor mRNA levels. Further, this decrease could be explained by the fact that GPHs can inhibit intestinal cholesterol absorption by suppressing the micellar solubility of cholesterol.^50^ This disruption of cholesterol micelles could be due to the abundance of hydrophobic peptides and/or amino acid residues present in the GPHs.

In our experimental conditions, despite the low serum cholesterol, FLUV, UGP and GPHs that administered for 10 weeks to rats fed HFFD increased markedly the HDL cholesterol levels. These results could suggest that UGP and GPHs enhanced efficiently cholesterol flow from peripheral tissues to plasma *via* the HDL involving then an efficient reverse cholesterol transport through the hepatobiliary pathway.

The overall results indicate that GPH-TF is more efficient in the protection against hyperlipidemia. The effect of GPH-TF in lowering of lipid levels might be explained by the presence of some potent little size bioactive peptides (DH 23%), which can provide beneficial action in HFFD rats.

These results are corroborated with numerous studies, which revealed that the administration of fish protein hydrolysates from *Boops boops*,^32^*Salaria basilisca*^51^ and *Sardinella aurita*^31^ improved significantly lipid profile of hyperlipidemic rats. Hosomi *et al.*^48^ and Liaset *et al.*^49^ also reported that the fish proteins are effective in reducing LDL-C concentration in hypercholesterolemic rats.

In humans, the normal reference values for atherogenic index (AI) and coronary risk index (CRI) should not be higher than 4 and 2.5, respectively.^52^ Thus, patients with cardiac risk indices higher than these reference values are predisposed to developing ischaemic heart disease and thrombotic cardiovascular accident.^53^ Thus, the high serum LDL-c and TC levels and low HDL-c level allowed assessing the lipid atherogenesis and considered a major risk factor for the development of insulin resistant, atherogenic risk and metabolic syndrome. Indeed, the excess of TC and LDL-c promotes accumulation of lipid in the arterial intima, thereby inducing the reduction of the diameter of the artery. This phenomenon increases the thrombosis risk. Thus, the investigation of the AI, the atherogenic index of plasma (AIP) and CRI were conducted in this study. The AI, defined as the ratio of LDL-c and HDL-c, was 3.6-fold higher in the HFFD group than the control group (Table 4). The hypercaloric diet induced also an increase of the AIP (log(TG/HDL-c)) and CRI (TC/HDL-c) by 1.6-fold and 1.8-fold, respectively. The rise of the AI, AIP and CRI risk has been significantly (*p* < 0.05) moderated and reduced to the normal values with the FLUV, as well as UGP and GPHs treatments.

The results suggest that the hydrolysis of goby proteins promote the generation of bioactive peptides which may regulate efficacy the lipid profile, and lowered in consequence the risk of atherogenic and coronary disease. This observation indicates that GPHs might have strong cardioprotective potential.

### 3.4. Effects of UGP and GPHs on hepatic TC and TG contents

Lipid content in liver tissues was also analyzed and the findings are shown in Table 5. A significant increase in liver TC and TG levels by approximately 58% and 149%, respectively, were observed in the rats fed hypercaloric diet compared to those of the control group (*p* < 0.05). The orally administration of UGP to rats fed HFFD significantly reduced (*p* < 0.05) the hepatic TG and TC values by about 21.23% and 42.59%, respectively, compared to those of the HFFD group. Further, the administration of GPHs as well as FLUV tends to significantly restore the levels of hepatic fats compared to HFFD group and the values obtained were even lower than those of rats treated with UGP (*p* < 0.05).

**Table 5:** Effects of UGP and GPHs on the levels of hepatic TC and TG^a^

	CD	HFFD	HFFD + FLUV	HFFD + UGP	HFFD + GPH-A	HFFD + GPH-TF
Liver (μmol g^−1^ tissue)						
TG	4.41 ± 0.11	6.97 ± 0.35^a^	4.57 ± 0.15^b^	5.49 ± 0.42^b^	3.91 ± 0.53^b^	4.75 ± 0.15^b^
TC	6.92 ± 0.42	17.21 ± 0.51^a^	8.16 ± 0.50^a,b^	9.88 ± 1.27^b^	9.20 ± 0.79^b^	9.52 ± 1.04^b^

a GPH-A and GPH-TF represent goby protein hydrolysates produced using bacterial proteases of *B. mojavensis* A21 and digestive enzymes from triggerfish (*Balistes capriscus*), respectively. UGP is the undigested goby proteins. Data are expressed as mean ± SEM (*n* = 6). ^a,b^ Indicate significant differences compared to the values of CD, and HFFD groups, respectively, at *p* < 0.05.

So, all of these results suggest that GPHs possess a potential lipid-lowering effect in plasma as well as the liver tissue, even after a long term of hypocaloric feed consumption. Several works also reported that protein hydrolysates from salmon^35^ and Alaska pollock^48^ have a potential to prevent liver lipid accumulation in rats fed high-cholesterol-diet. Overall, the obtained results are in agreement with several works which shown the benefic effects of the administration of fish protein hydrolysates to hypolipidemic rats.^31,32,48,49^

These observations indicated that the GPHs and the UGP, in particular the GPH-TF, might have a strong cardioprotective effect similar to fluvastatin drug which is recommended to treat high cholesterol in adults and children.

### 3.5. Effects of UGP and GPHs on pancreatic lipase activity

**Fig. 3:**
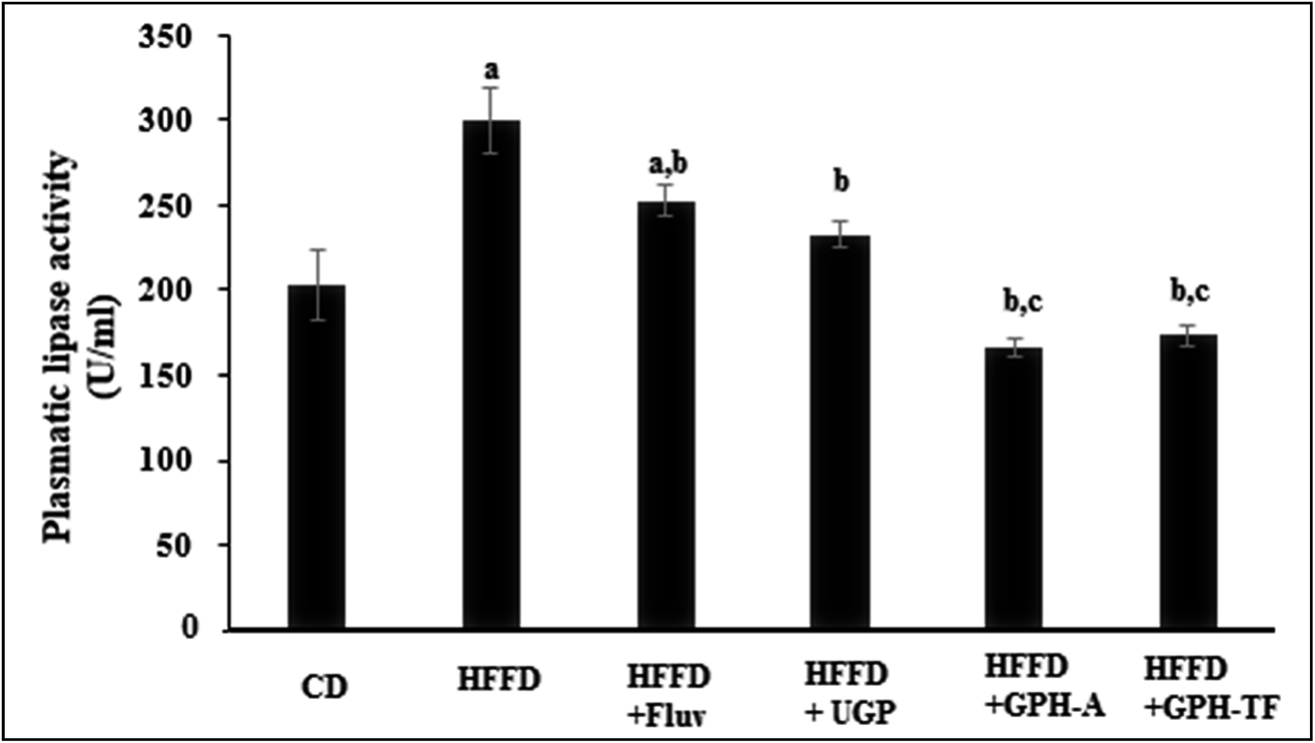
Effects of UGP and GPHs administration on lipase hepatic activity. GPH-A and GPH-TF represent goby protein hydrolysates produced using bacterial proteases of *Bacillus mojavensis* A21 and digestive enzymes from triggerfish (*Balistes capriscus*), respectively. UGP is the undigested goby proteins. Data are expressed as mean ± SEM (*n* = 6). ^a,b,c^ Indicate significant differences compared to the values of CD, HFFD and HFFD + FLUV groups, respectively, at *p* < 0.05.

Obviously, in humans and in animal models, the consumption of hypercaloric diet for a long period induces a chronic obesity state. Drug's control in lipid metabolism offers an efficient pathway to prevent populations against dyslipidemia risk. Several enzymes involved in lipid metabolism have yielded a rich pool of these drugs for treating overweight and metabolic disorders,^54^ and the pancreatic lipase inhibitors represent one of the most prescribed drugs. Besides its role as a hydrolytic enzyme of dietary fats,^55^ pancreatic lipase is also considered as the first responsible of the absorption of gastrointestinal triglycerides. The enzyme hydrolysis phospholipids and triacylglycerols and plays an important role in the metabolic processing of LDL-c and HDL-c.^44^ Thus, pancreatic lipase's inhibition is considered as an effective approach for the treatment of dyslipidemia. The pancreatic lipase (PL) activities of the different experimental rats are illustrated in Fig. 3. After 10 weeks of treatment, the PL activity in rats fed HFFD was increased significantly by 30.35% (*p* < 0.05) as compared to the control group. The administration of FLUV and UGP decreased activity of PL by 15.61% and 22.30%, respectively, as compared to HFFD group (*p* < 0.05). However, the obtained values were still higher than that of CD group. Interestingly, the administration of GPH-A and GPH-TF significantly decreased PL activities by 44.53% and 42.08% in comparison with that of the HFFD group (*p* < 0.05), and the obtained values were even lower than that of the control group. The results demonstrate once again the importance of *in vitro* protein hydrolysis. Similar results were also reported by Ktari *et al.*^51^ who showed that zebra blenny protein hydrolysates could reduce lipid accumulation by an inhibition of the PL activity.

Therefore, the investigation of inhibitory effect of lipase activity suggested that goby proteins and both hydrolysates might induce a hypolipidemic effect through the suppression of lipogenesis, lipid absorption and digestion and could therefore prevent risk of coronary artery disease.^56^ Indeed, the pancreatic lipase cleaves the dietary triglycerides to a monoacyl glycerides and free fatty acids.^57^ Thus, the inhibition of PL activity leads to decrease in intestinal lipid digestion and absorption by enterocytes, and increase their excretion in the feces and thereby energy uptake, which is one of the key targets to mediate dyslipidemia.

### 3.6. Effects of UGP and GPHs on coagulation time

Clotting tests, activated partial thromboplastin time (aPTT) and prothrombin time (PT), are commonly used in clinical biochemistry to evaluate the performance of the intrinsic and extrinsic coagulation factor, respectively. The effect of UGP and its hydrolysates in different stages of the coagulation cascade *ex vivo* are presented in Table 6. Regarding PT, a significant increase in the prothrombin time by 29% in serum of rats fed HFFD were depicted compared to the CD group.

**Table 6:** Clotting tests: Prothrombin time (PT) test and activated partial thromboplastin time (aPTT) test^a^

	CD	HFFD	HFFD + FLUV	HFFD + UGP	HFFD + GPH-A	HFFD + GPH-TF
aPTT (sec)	107.39 ± 8.48	44.72 ± 2.31^a^	115.67 ± 2.60^b^	118.00 ± 2.80^b^	114.66 ± 4.46^b^	115.67 ± 3.01^b^
PT (sec)	36.65 ± 1.69	47.21 ± 2.52	31.30 ± 4.77^b^	12.19 ± 1.63^a,b,c^	17.74 ± 5.00^a,b,c^	22.58 ± 3.10^a,b^

a GPH-A and GPH-TF represent goby protein hydrolysates produced using bacterial proteases of *B. mojavensis* A21 and digestive enzymes from triggerfish (*Balistes capriscus*), respectively. UGP is the undigested goby proteins. Data are expressed as mean ± SEM (*n* = 6). ^a,b,c^ Indicate significant differences compared to the values of CD, HFFD and HFFD + FLUV groups, respectively, at *p* < 0.05.

On the other hand, the results obtained from aPTT indicate that the hypercaloric diet reduces the plasma clotting time of rats as compared to the normal control group (44.72 s *vs.* 107.39 s). Therefore, the reduction of plasma clotting time and the hypersecretion of prothrombin induced by high fat and fructose diet, could lead to an increase the risk of thrombosis in untreated group compared to CD group. Tholstrup *et al.*^58^ reported that the high fatty acid diet, especially the stearic acid, increases the risk of developing thrombosis and myocardial disease, which might be explained by the increase of the Factor VII expression. It is interesting to note that the administration of fluvastatin, undigested proteins and GPHs could repair the disruption caused by the high calorie diet. Results illustrated in Table 6, clearly demonstrated that the thrombin clotting time (aPTT) of all treated rats were significantly lengthened compared to the HFFD rat, and were similar to that of CD rats.

Regarding PT assay, the HFFD + FLUV and HFFD + GPH-TF groups showed markedly lower PT (33.70 and 52.17%, respectively) than did the untreated HFFD rats. Indeed, the administration of UGP and GPH-A were efficient to reduce PT levels compared to that of HFFD group, and even more lower than the CD group by 66.63 and 51.60%, respectively (*p* < 0.05).

### 3.7. Histological examination of liver

**Fig. 4:**
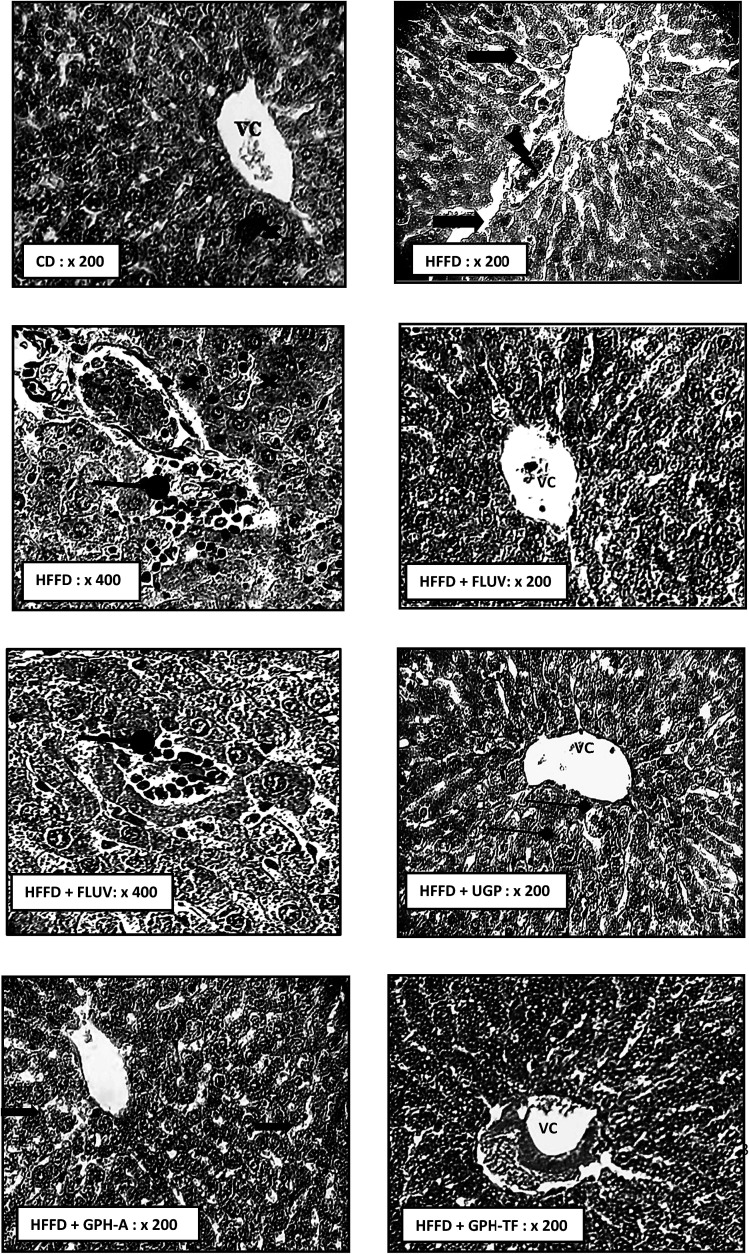
Effects of UGP and GPHs administration on liver histopathology (H–E staining) in hypercaloric rats induced by high-fat/fructose diet. High-fat/fructose-diet HFFD diet without goby protein supplementation (HFFD), high-fat/fructose-diet with fluvastatin supplementation of 20 mg kg^−1^ BW (HFFD + FLUV), high-fat/fructose-diet with UGP supplementation of 400 mg kg^−1^ BW (HFFD + UGP), high-fat/fructose-diet with GPH-A supplementation of 400 mg kg^−1^ BW (HFFD + GPH-A) and high-fat/fructose-diet with GPH-TF supplementation of 400 mg kg^−1^ BW (HFFD + GPH-TF). 
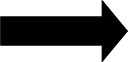
 lipid vacuolization; 
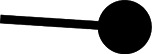
 leukocyte infiltrations; 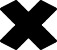 cells necrosis; 
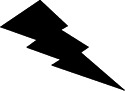
 vascular congestion; 
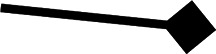
 mitotic cell.

Dyslipidemia is associated to histological changes in liver among the rat groups. As well-known, liver is the most important organ for lipids and cholesterol metabolism in order to maintain normal body functions.^59^ The biochemical findings were supported by the histo-pathological observations. Indeed, Fig. 4 H&E stained HFFD rat liver section revealed serious deterioration of hepatocytes with cell necrosis and leukocytes infiltration, vascular congestion and the loss of architecture. Moreover, hypercaloric diet induced severe fatty changes in hepatocytes. This effect may be due to the deposition of fat droplets in hepatocytes. Thus, histological results of HFFD rat liver could be attributed to the oxidative stress, which usually damages cell function and lead to cell death by necrosis or apoptosis. Interestingly, the Fluv, UGP and GPH-TF treatments prevented liver structure damage. These results indicate the effectiveness of goby fish peptides in the prevention against lipid accumulation in liver. The liver section of treated rat GPH-A showed a reduction of lipid accumulation and an amelioration in hepatocyte architecture. All of above histopathological observations were in accordance with biochemical findings, and further approved the hepatoprotective and hypolipidemic potential of GPHs.

## 4. Conclusion

Goby protein hydrolysates demonstrated an interesting ability to reduce hyperlipidemia by decreasing the serum levels of TC, TG and LDL-c, and increasing the level of HDL-c in rats fed high fat and fructose diet. The hypolipidemic effect of GPH-TF was greater than did GPH-A and even better than FLUV. Additionally, the reduction of atherogenic indexes in rats treated with GPH-TF revealed its ability as a cardioprotective agent. Thus, the enzymatic hydrolysis is an efficient process for bioactive peptides production, which exhibited cardiovascular activity more prominent than the undigested proteins. The overall results demonstrate that GPH-TF could be an excellent dietary supplement preventing against hyperlipidemia and obesity.

## Conflicts of interest

The authors declare that they have no conflict of interest.

## Supplementary Material
